# A comparative study of different methods for the determination of cadmium in various tissues of ramie (*Boehmeria nivea* L.)

**DOI:** 10.1007/s10661-023-11601-2

**Published:** 2023-07-31

**Authors:** Kunmei Chen, Pan Mou, Aiguo Zhu, Ping Chen, Jikang Chen, Gang Gao, Xiaofei Wang, Xinkang Feng, Chunming Yu

**Affiliations:** Institute of Bast Fiber Crops, Chinese Academy of Agricultural Sciences, Changsha, 410205 China

**Keywords:** Cadmium determination, Plant sample, Method comparison, Cadmium stress, Remediation of Cd pollution

## Abstract

Remediation of cadmium (Cd) pollution is one of the priorities of global environmental governance and accurate detection of Cd content is a key link in remediation of Cd pollution. This study aimed to compare three methods (inductively coupled plasma optical emission spectrometry (ICP-OES), inductively coupled plasma mass spectrometry (ICP-MS), and graphite furnace-atomic absorption spectrometry (GF-AAS)) for the determination of Cd with different tissues of various ramie varieties, and distinguish the advantage and disadvantage of each method. In total, 162 samples of ramie (*Boehmeria nivea* L.), which is an ideal plant for heavy metal remediation, were detected and the results showed that the three methods were all suitable for the de-termination of Cd content in ramie. ICP-OES and ICP-MS were simpler, faster, and more sensitive than GF-AAS. ICP-MS could be recommended for the determination of samples with various concentrations of Cd. ICP-OES could be used for measurement of samples with > 100 mg/kg Cd content, while GF-AAS was suitable for the detection of samples with very high (> 550 mg/kg) or very low (< 10 mg/kg) Cd content. Overall, considering the accuracy, stability, and the cost of measurement, ICP-MS was the most suitable method for determination of Cd content. This study provides significant reference information for the research in the field of Cd pollution remediation.

**Supplementary Information:**

The online version contains supplementary material available at 10.1007/s10661-023-11601-2.

## Introduction

Cadmium (Cd) is a toxic heavy metal that has a great potential risk to human health. Long-term exposure to cadmium can damage humans’ reproductive system, muscles, and bones, to a significant extent, related to various types of cancer (de Angelis et al., 2017; Kumar & Sharma, 2019; Reyes-Hinojosa et al., 2019). Due to mining activities, the soil in mineral areas has higher Cd concentration than that in other regions. Rice grown up on high Cd–contaminated soil has higher Cd concentration than that in the standard level (Du et al., 2013). Cd pollution was characterized in the area of the mining at Guiyang, northeast of Hunan Province in China, and it showed a high ecological risk (Lu et al., 2015). Another study found that Cd mainly comes from agriculture activities (Zhang et al., 2020). Based on the Cd contamination current situation, researchers devoted themselves to remediation of soil heavy metal pollution. Many researches showed that bioremediation is an eco-friendly and efficient method of reclaiming environments contaminated with heavy metals by making use of the inherent biological mechanisms of microorganisms and plants to eradicate hazardous contaminants (Ojuederie & Babalola, 2017). Phytoremediation is an emerging technology implementing green plants to clean up the environment from contaminants and has been considered as a cost-effective and non-invasive alternative to the conventional remediation approach (Ashraf et al., 2019). Whether it is the evaluation of soil Cd pollution degree, food safety, human health, or the evaluation of phytoremediation Cd pollution effect, it is necessary to detect the Cd content of corresponding samples. An effective, rapid, and accurate measurement method of Cd content is an important link in the field of Cd pollution control. Ramie (*Boehmeria nivea* L.) has a long-time planting history in China, which is a high-quality natural fiber crop and has potential for remediation of heavy metal–contaminated soils (Sun et al., 2014; Yang et al., 2010). It is known that ramie has high tolerance to Cu, Cd, Pb, and Zn, as well as high accumulation (Lan et al., 2020; Rehman et al., 2019; She et al., 2018). Many researches were performed on the effects of chelators, peat, abscisic acid, and biochar to repair Cd pollution in ramie (Chen et al., 2021; Gong et al., 2019). In that context, how to quickly and accurately measure the Cd content in ramie is very important. The analytical precision of Cd determination plays a special role in the remediation effect on heavy metal–contaminated soil.

There are many methods for determination of Cd content. For example, the molybdenum-coated T-shaped slotted quartz tube atom trap flame atomic absorption spectrophotometry method (Kasa et al., 2020) is considered a sensitive and accurate way to measure Cd concentration in foods. The proposed method based on “turn-on” fluorescence of NP-1 is simple, sensitive, and reliable for rapid determination of Cd in samples with high applicability and stability (Tumay et al., 2020). A method for the determination of Cd in herb samples based on solidified floating organic drop microextraction (SFODME) using 1-(2-pyridylazo)-2-naphthol (PAN) as a chelating reagent and detection by electrothermal atomic absorption spectrometry (ETAAS) is developed rapidly (Thongsaw et al., 2017). Determination of Cd in fish by atomic absorption spectrometry with electrothermal atomization is based on sample digestion in a microwave oven and subsequently read using an atomic absorption spectrometer with a graphite furnace (Costa et al., 2012). Determination of Cd in bread and biscuit samples using ultrasound-assisted temperature-controlled ionic liquid microextraction is presented as a simple, cheap, ecological, and sensitive alternative (Santos et al., 2019). An all-solid light addressable potentiometric sensor (LAPS) is presented for determination of Cd in rice, which is satisfactory precision, accuracy, and selectivity (Zhang et al., 2018). An immunochromatography kit was used to determination Cd in rice, which is an inexpensive, reliable tool for quick and easy on-site determination of Cd in cereals and soybeans (Abe et al., 2014). One research showed that different elements have their own most suitable detection methods, such as for Pb, the most suitable method is ICP-MS, and for Zn, the most suitable method is AAS (Pan et al., 2020). Thus, which is the most suitable method for Cd determination is worth to be considered. ICP-OES and ICP-MS were used for the analysis of heavy metals in leaves, fruits, and branches of mistletoe (Kamar et al., 2018), and also used for precise measurement of major and trace elements in bulk pyrite and magnetite (Liu et al., 2020), essential and non-essential/toxic trace metals in the edible parts of some common vegetables (Iftikhar-Ul-Haq et al., 2021), and measure trace elements in baby food, milk power, and inorganic contaminants (Kiani et al., 2022; Krzyzaniak et al., 2019). GF-AAS coupled with the method of mild extraction using diluted acid is an efficient, cost-saving, convenient and friendly way to measure Cd concentration in grain (Zhou et al., 2019). ICP-MS and GF-AAS were useful and fast methods for blood lead and Cd determination (Trzcinka-Ochocka et al., 2016). ICP-OES is a common method for determination of soil Cd (McBride, 2011). ICP-OES, GF-AAS, and ICP-MS are widely used and cost effective, but which one is the best way to determination of Cd and which one is faster and more accurate should be discussed. In this study, the three widely used methods (ICP-OES, ICP-MS, GF-AAS) were compared with various tissues (root, stem, leaf) from different varieties of ramie, which were grown under Cd conditions. And the advantage and disadvantage for each method were discussed.

## Materials and methods

### Ethics statement

The ramie varieties Zhongzhu No. 1 (zz1), Zhonngzhu No. 3 (zz3), and Zhongzhu No. 4 (zz4) used in this study were bred by the Institute of Bast Fiber Crops, Chinese Academy of Agricultural Sciences, China. Therefore, no specific permissions were required for using these specimens. All methods comply with relevant institutional, national, and international guidelines and legislation.

### Plant materials and Cd treatment experiment

Three ramie varieties Zhongzhu No. 1 (zz1), Zhonngzhu No. 3 (zz3), and Zhongzhu No. 4 (zz4) were used in this study, which were bred by the Institute of Bast Fiber Crops Chinese Academy of Agricultural Sciences. The lateral branches of ramie plants were sampled and cultured by hydroponic culture (Chen et al., 2018). Meanwhile, we dried the soil and divided it into six equal portions, then sprayed it with different concentrations of cadmium chloride, making sure the Cd concentrations of six portions were 0, 10, 30, 50, 80, and 150 mg/kg, respectively. Finally, we weighed 12 kg of the soil in a pot. Each treatment was set for three repetitions. The lateral branches with 10-cm roots were planted in the pot, one plant in one pot. The pots were put in a greenhouse under the following conditions: 500–600 μmol m^-2^ s^-1^ light, 12-h light/12-h dark period, 25–30°C, and 60–70% relative humidity. After 3 months of cadmium stress, root, stem, and leaf in each pot were harvested, blanked at 105°C for 30 min, dried to constant weight at 75°C, and ground into powder.

### Sample preparation

All reagents used in this study were at least of analytical grade. The Cd standard solutions were provided by the internet of National Standard Material Center and National Nonferrous Metals and Electronic Materials Analysis and Testing Center (Beijing, China).

Preparation of Cd standard solution for ICP-OES: 2.5 mL of 1000 μg/ml Cd standard solution was added to 25-mL volumetric flask and diluted to the mark with 1% nitric acid to prepare 100 mg/L Cd intermediate solution. Then 0 mL, 0.25 mL, 0.75 mL, and 1.25 mL Cd intermediate solutions were added to a 25-mL volumetric flask and diluted to the mark with 1% nitric acid, making the standard series concentrations of 0, 1, 3, and 5 mg/L, respectively.

Preparation of Cd standard solution for ICP-MS: 5 mL of 1000 μg/mL Cd standard solution was added to a 50-mL volumetric flask and diluted to the mark with ultrapure water to prepare 100 mg/L Cd intermediate solution. In total, 0.5 mL Cd intermediate solution was absorbed to a 100-mL volumetric flask and diluted to the mark with ultrapure water. Then, 1, 2, 4, and 10 mL of 0.5 mg/L Cd solution was added to a 100-mL volumetric flask and diluted to the mark with ultrapure water, making the standard series concentrations of 5, 10, 20, and 50 μg/L, respectively. Finally, 0, 2, 5, 10, and 20 mL of the 10 μg/L Cd solution was added to a 100-mL volumetric flask and diluted to the mark with ultrapure water. The final Cd standard solution gradients were 0, 0.2, 0.5, 1, 2, 5, 10, 20, and 50 μg/L.

The method of preparing the Cd standard solution for GF-AAS was the same as the method for ICP-MS, and the final Cd standard solution gradients were 0, 0.2, 0.5, 1, 2, and 2.5 μg/L.

Before Cd measure, the plant sample was prepared with 65% HNO_3_. Teflon reaction vessels were used in all digestion procedures. For microwave-assisted digestion, 0.1 g of dried ramie (in triplicate) was weighted and 10 mL HNO_3_ was added to each Teflon flask (Pan et al., 2018). Ramie sample and nitric acid were homogenized using vortex, put in the closed microwave digestion system, and digested with the program showed in Table S1. After digestion, the capsule was opened and heated to dryness gently. When 1~2 mL liquid was left in the capsule stopped heating, it was transferred to a 25-mL volumetric flask. Then the capsule was washed with ultrapure water for several times, and finally diluted to the mark. Three replicates were set for each sample.

### Determination of Cd content

Cd content was measured by ICP-OES (plasma atomic emission spectrometer ICPE-9820, SHIMZDZU), GF-AAS (iCE3500 AA Atomic absorption spectrometer), and ICP-MS (iCAP Q MS, Thermo scientific), respectively. ICP-OES instrumental conditions are listed in Table S2. The operating parameters of the ICP-MS are shown in Table S3. The data acquisition mode was full quantitative, and the determination was repeated three times. The program of GF-AAS is listed in Table S4.

### Statistical analysis

Statistical analysis was performed using IBM SPSS Statistics 25. The Kolmogorov-Smirnov and Shapiro-Wilk tests were used to determine the normality of each dataset. The Wilcoxon signed-rank test was used to determine statistical similarities or differences for non-parametric datasets containing two related samples. Sample linear regression analysis and correlation were conducted using GraphPad Prism 9.

## Results

### Evaluation of standard curve

The limit of quantitation (LOQ) and limit of detection (LOD) of cadmium content measured by ICP-MS, ICP-OES, and GF-AAS are listed in Table 1. The ultrapure water was used as a blank solution through the whole process of the three methods. Three times standard deviation of 10 consecutive measurements was used as the value of LOD, and 10 times of the standard deviation was used as the value of LOQ (D'Archivio et al., 2019; Iftikhar-Ul-Haq et al., 2021; Peng & Liu, 2019). The lower values of detection limits indicated that the three methods provided adequate sensitivity.

**Table 1:** The standard curve limit of detection and limit of quantification of the ICP-OES, ICP-MS, and GF-AAS methods

Method	Linear equation	Correlation coefficient *R*^2^	LOD (mg/L)	LOQ (mg/L)
GF-AAS	*y*=0.11759*x*+0.0176	0.99210	0.00845	0.0338
ICP-MS	*y*=5.985*x*+31.644	1.00000	0.00041	0.0016
ICP-OES	*y*=(9.2783e−005)*x*−0.0396	0.99997	0.34990	1.3996

### Comparison of Cd content determined by GF-AAS, ICP-MS, and ICP-OES

**Fig. 1:**
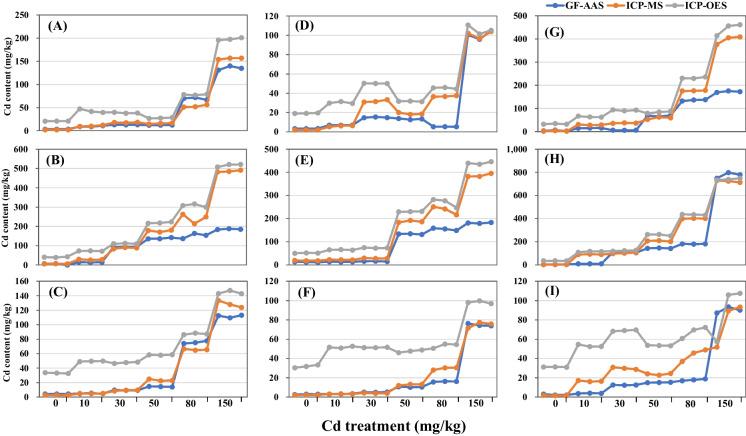
Cd content of roots, stems, and leaves of ramie determined by GF-AAS, ICP-MS, and ICP-OES. **A–C** The Cd content of roots, stems, and leaves in Zhongzhu No. 1. **D–F** The Cd content of roots, stems, and leaves in Zhongzhu No. 3. **G–I** The Cd content of roots, stems, and leaves in Zhongzhu No. 4

To receive the detected samples, ramie plants were set to Cd stress with various Cd concentrations. In total, 162 samples including 54 stems, 54 leaves, and 54 roots of three ramie varieties were used for Cd content determination. We observed that the Cd contents for the three detected ramie varieties were about 1–160 mg/kg in leaf, 1–500 mg/kg in root, and 1–800 mg/kg in stem (Fig. 1). To evaluate the precision and stability of the instruments, the relative standard deviation (RSD) was analyzed using these data measured by GF-AAS, ICP-MS, and ICP-OES. The results showed that the RSDs of roots, stems, and leaves in the three detected ramie varieties were almost all less than 5% (Table 2), which indicated that accurate data can be obtained by using these three instruments to detect Cd content in a large range (1–800 mg/kg).

**Table 2:** The relative standard deviation (RSD) of cadmium content in root of ramie measured by ICP-OES, ICP-MS, and GF-AAS methods

Cd treatment (mg/kg)	Variety	GF-AAS			ICP-MS			ICP-OES		
		Root	Stem	Leaf	Root	Stem	Leaf	Root	Stem	Leaf
0	zz1	2.838	4.318	0.719	3.334	5.812	4.175	0.657	3.728	1.372
	zz3	1.862	2.429	5.300	1.120	3.726	2.101	1.349	1.319	3.858
	zz4	1.160	1.460	2.595	0.567	6.054	6.800	4.184	0.657	0.451
10	zz1	0.094	4.599	4.798	2.036	5.828	1.764	2.742	1.057	1.724
	zz3	1.006	2.917	4.346	0.750	2.216	0.966	2.676	1.189	2.382
	zz4	2.430	0.217	4.572	4.222	1.402	2.875	2.927	3.040	1.968
30	zz1	1.349	0.708	2.494	4.841	3.302	6.070	2.348	0.720	0.583
	zz3	0.527	4.136	2.660	4.277	3.072	4.958	0.794	1.359	0.236
	zz4	3.059	1.808	0.956	2.420	3.592	3.218	1.957	2.525	0.299
50	zz1	0.710	2.070	2.068	2.081	2.452	4.521	2.404	1.416	0.503
	zz3	3.608	1.079	2.073	3.203	1.888	4.661	1.407	0.353	1.465
	zz4	3.260	1.464	1.165	2.865	1.677	3.591	4.308	2.311	7.292
80	zz1	3.003	2.963	2.036	4.304	2.681	1.147	1.012	2.192	0.980
	zz3	2.207	2.648	1.642	1.334	6.216	3.760	0.240	5.740	3.725
	zz4	1.909	0.565	4.207	0.639	0.364	2.759	1.180	0.520	1.066
150	zz1	2.717	0.918	1.376	0.827	0.803	3.051	1.099	1.203	1.423
	zz3	3.577	0.914	1.382	3.067	1.588	3.379	3.587	0.991	1.204
	zz4	1.413	2.620	2.810	3.634	0.943	2.199	4.767	0.787	0.770

In fact, when RSD was below 20%, it can be considered an acceptable limit for ICP-OES analysis in some complex samples (Altundag & Tuzen, 2011; D'Archivio et al., 2019; Dolan & Capar, 2002). Thus, the small accidental error for the Cd content determination measured by GF-AAS, ICP-MS, and ICP-OES in our study suggested that the three methods were feasible for Cd determination in ramie. Obviously, the value measured by ICP-OES for each sample was higher than that detected by GF-AAS and ICP-MS (Fig. 1), which may be caused by its high sensitivity. For samples with Cd content less than 100 mg/kg, the values detected by GF-AAS and ICP-MS were relatively close, especially when the Cd content was less than 10 mg/kg; there was almost no difference between the values measured by these two methods. To compare the differences of the values detected by the three methods, significance analysis was performed using SPSS with one-way ANOVA mode. The result showed that when the Cd content of the samples was about 10 mg/kg, and there was no significant difference between the values detected by GF-AAS and ICP-MS in roots, stems, and leaves of zz1 (Fig. 1, Table 3).

**Table 3:** Significance analysis of Cd content determined by GF-AAS, ICP-MS, and ICP-OES in Zhongzhu No. 1

Cd treatment (mg/kg)	Comparison	Root		Stem		Leaf	
		MD	*p*-value	MD	*p*-value	MD	*p*-value
0	A-M	1.226	0.000	−1.445	0.533	1.484	0.002
	A-O	−17.474	0.000	−36.416	0.000	−28.913	0.000
	M-O	−18.701	0.000	−34.972	0.000	−30.397	0.000
10	A-M	−0.946	0.664	−13.538	0.000	0.186	0.434
	A-O	−33.230	0.000	−58.212	0.000	−44.479	0.000
	M-O	−32.285	0.000	−44.674	0.000	−44.665	0.000
30	A-M	−4.837	0.000	6.026	0.015	0.196	0.750
	A-O	−25.709	0.000	−17.122	0.000	−38.117	0.000
	M-O	−20.872	0.000	−23.148	0.000	−38.314	0.000
50	A-M	−3.540	0.001	−38.629	0.000	−9.019	0.000
	A-O	−15.289	0.000	−81.303	0.000	−43.886	0.000
	M-O	−11.749	0.000	−42.674	0.000	−34.866	0.000
80	A-M	16.354	0.000	−90.668	0.001	9.982	0.000
	A-O	−8.439	0.004	−156.631	0.000	−11.581	0.000
	M-O	−24.793	0.000	−65.963	0.003	−21.563	0.000
150	A-M	−20.652	0.000	−300.270	0.000	−16.753	0.001
	A-O	−62.814	0.000	−330.800	0.000	−32.671	0.000
	M-O	−42.162	0.000	−30.530	0.000	−15.918	0.001

*A-M*, *A-O*, and *M-O* denote the differences of the average values determined by GF-AAS and ICP-MS, GF-AAS and ICP-OES, and ICP-MS and ICP-OES, respectively. *MD* denotes mean difference

When the Cd content of the sample was greater than 10 mg/kg but less than 100 mg/kg, although the values detected by GF-AAS and ICP-MS have reached a significant level, the difference was much less than that detected by ICP-OES. For example, under the treatment of 50 mg/kg Cd concentration, the difference between the values detected by GF-AAS and ICP-MS in roots of zz1 was only −3.540. Similar results were observed in roots, stems, and leaves of zz3 and zz4 (Fig. 1, Table 4 and Table 5), which further confirmed that GF-AAS and ICP-MS were more suitable for the determination of samples with Cd content less than 100 mg/kg when comparing with ICP-OES. When the Cd content of the sample was 100–550 mg/kg, the values detected by ICP-MS and ICP-OES were close, while the values detected by GF-AAS were significantly lower than those detected by the other methods (Fig. 1; Tables 3, 4, 5). When the Cd content of the sample was greater than 550 mg/kg, the values detected by the three methods showed little difference, which was also confirmed by the results of difference analysis (Fig. 1; Tables 3, 4, 5). These results suggested that ICP-MS and ICP-OES were more suitable for the determination of samples with Cd content of about 100–550 mg/kg, while the three methods were all suitable in measuring samples with Cd content greater than 550 mg/kg. Overall, our study suggested that ICP-MS was applicable to samples with various concentrations of Cd, and ICP-OES could be used for measurement of samples with > 100 mg/kg Cd content, while GF-AAS was suitable for the detection of samples with very high (> 550 mg/kg) or very low (< 10 mg/kg) Cd content.

**Table 4:** Significance analysis of Cd content determined by GF-AAS, ICP-MS, and ICP-OES in Zhongzhu No. 3

Cd treatment (mg/kg)	Comparison	Root		Stem		Leaf	
		MD	*p*-value	MD	*p*-value	MD	*p*-value
0	A-M	1.363	0.000	–6.063	0.000	0.692	0.370
	A-O	−15.940	0.000	−38.701	0.000	−28.956	0.000
	M-O	−17.303	0.000	−32.638	0.000	−29.648	0.000
10	A-M	0.985	0.126	−8.692	0.000	0.127	0.785
	A-O	−23.228	0.000	−51.575	0.000	−48.374	0.000
	M−O	−24.213	0.000	−42.883	0.000	−48.501	0.000
30	A-M	−16.866	0.000	−12.967	0.000	1.032	0.001
	A-O	−35.211	0.000	−57.906	0.000	−46.330	0.000
	M-O	−18.345	0.000	−44.939	0.000	−47.361	0.000
50	A-M	−5.576	0.000	−54.102	0.000	−2.374	0.019
	A-O	−18.370	0.000	−96.929	0.000	−37.045	0.000
	M-O	−12.794	0.000	−42.826	0.000	−34.672	0.000
80	A-M	−31.530	0.000	−82.243	0.001	−13.554	0.000
	A-O	−40.041	0.000	−114.669	0.000	−37.291	0.000
	M-O	−8.511	0.000	−32.426	0.041	−23.737	0.000
150	A-M	−0.526	0.886	−205.398	0.000	−0.082	0.963
	A-O	−5.358	0.178	−258.831	0.000	−23.421	0.000
	M-O	−4.832	0.218	−53.433	0.000	−23.338	0.000

*A-M*, *A-O*, and *M-O* denote the differences of the average values determined by GF-AAS and ICP-MS, GF-AAS and ICP-OES, and ICP-MS and ICP-OES, respectively. *MD* denotes mean difference

**Table 5:** Significance analysis of Cd content determined by GF-AAS, ICP-MS, and ICP-OES in Zhongzhu No. 4

Cd treatment (mg/kg)	Comparison	Root		Stem		Leaf	
		MD	*p*-value	MD	*p*-value	MD	*p*-value
0	A-M	1.615	0.329	0.607	0.014	0.811	0.022
	A-O	−28.756	0.000	−31.566	0.000	−28.704	0.000
	M-O	−30.371	0.000	−32.173	0.000	−29.515	0.000
10	A-M	−13.951	0.000	−81.233	0.000	−12.661	0.000
	A-O	−48.876	0.000	−104.190	0.000	−49.217	0.000
	M-O	−34.925	0.000	−22.957	0.000	−36.557	0.000
30	A-M	−30.883	0.000	5.000	0.145	−17.383	0.000
	A-O	−86.280	0.000	−16.173	0.002	−56.458	0.000
	M-O	−55.398	0.000	−21.173	0.000	−39.075	0.000
50	A-M	10.064	0.026	−62.136	0.000	−8.640	0.000
	A-O	−15.186	0.004	−114.887	0.000	−38.323	0.000
	M-O	−25.250	0.000	−52.751	0.000	−29.683	0.000
80	A-M	−41.061	0.000	−219.658	0.000	−25.942	0.001
	A-O	−96.609	0.000	−253.348	0.000	−49.607	0.000
	M-O	−55.548	0.000	−33.690	0.000	−23.665	0.001
150	A-M	−224.559	0.000	53.417	0.006	12.046	0.511
	A-O	−271.574	0.000	34.700	0.035	−0.102	0.995
	M-O	−47.015	0.019	−18.717	0.194	−12.148	0.508

A-M, A-O, and M-O denote the differences of the average values determined by GF-AAS and ICP-MS, GF-AAS and ICP-OES, and ICP-MS and ICP-OES, respectively. MD denotes mean difference

### Correlation analysis of Cd content determined by GF-AAS, ICP-MS, and ICP-OES

To evaluate the relationships among the values detected by GF-AAS, ICP-MS, and ICP-OES, correlation analysis was performed. The result showed that, in roots of ramie, the correlation coefficients between GF-AAS and ICP-MS, GF-AAS and ICP-OES, and ICP-MS and ICP-OES were 0.899, 0.892 and 0.992, respectively (Table 6). In stems, the correlation coefficient of the pairwise comparison among the three methods was 0.873–0.997, while in leaves it was 0.907–0.969. Overall, the correlation coefficient between ICP-MS and ICP-OES in leaves (0.969), stems (0.997), and roots (0.992) was all the highest among the three pairwise comparisons. All these correlation coefficients abovementioned had reached significant level at p < 0.01, which suggested that the data obtained by the three methods have high stability and reliability.

**Table 6:** Correlation analysis of Cd content determined by GF-AAS, ICP-MS, and ICP-OES

		GF-AAS	ICP-MS	ICP-OES
Root	GF-AAS	1.000	0.899**	0.892**
	ICP-MS	0.899**	1.000	0.992**
	ICP-OES	0.892**	0.992**	1.000
Stem	GF-AAS	1.000	0.883**	0.873**
	ICP-MS	0.883**	1.000	0.997**
	ICP-OES	0.873**	0.997**	1.000
Leaf	GF-AAS	1.000	0.953**	0.907**
	ICP-MS	0.953**	1.000	0.969**
	ICP-OES	0.907**	0.969**	1.000

**Significantly different at the p < 0.01 level

### Regression analysis of Cd content determined by GF-AAS, ICP-MS, and ICP-OES

**Fig. 2:**
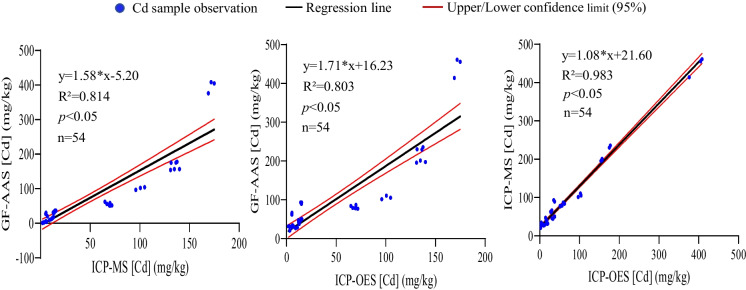
Regression analysis of Cd content in roots of ramie measured by ICP-MS, ICP-OES, and GF-AAS

**Fig. 3:**
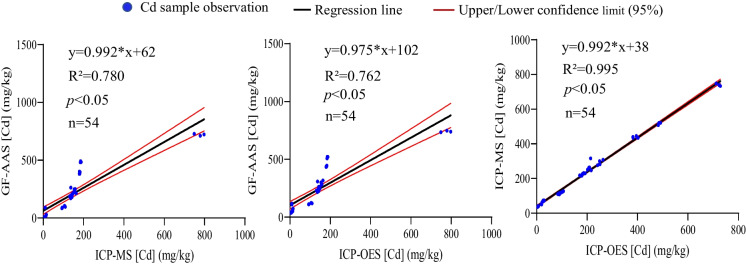
Regression analysis of Cd content in stems of ramie measured by ICP-MS, ICP-OES, and GF-AAS

**Fig. 4:**
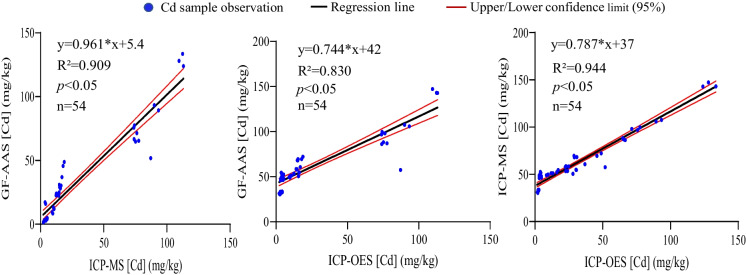
Regression analysis of Cd content in leaves of ramie measured by ICP-MS, ICP-OES, and GF-AAS

To further evaluate the relationships among the values detected by GF-AAS, ICP-MS, and ICP-OES, regression analysis was performed. The results showed that the regression coefficients (R^2^) between ICP-MS and GF-AAS, ICP-OES and GF-AAS, and ICP-MS and ICP-OES in roots were 0.814, 0.803, and 0.983, respectively (Fig. 2). In stems, the R^2^ between ICP-MS and GF-AAS, and ICP-OES and GF-AAS were 0.780 and 0.762, respectively, while the highest value of R^2^ (0.995) was in the comparison of ICP-MS and ICP-OES (Fig. 3). Strong relationships were also observed between ICP-MS and GF-AAS (R^2^=0.909), ICP-OES and GF-AAS (R^2^=0.830), and ICP-MS and ICP-OES (R^2^=0.944) in leaves (Fig. 4). These data suggested that whether in root, stem, or leaf of ramie samples, the comparison of ICP-MS and ICP-OES had a stronger relationship than the relationship between ICP-MS and GF-AAS, and also the relationship between GF-AAS and ICP-OES, which was consistent with the results of the correlation analysis.

**Fig. 5:**
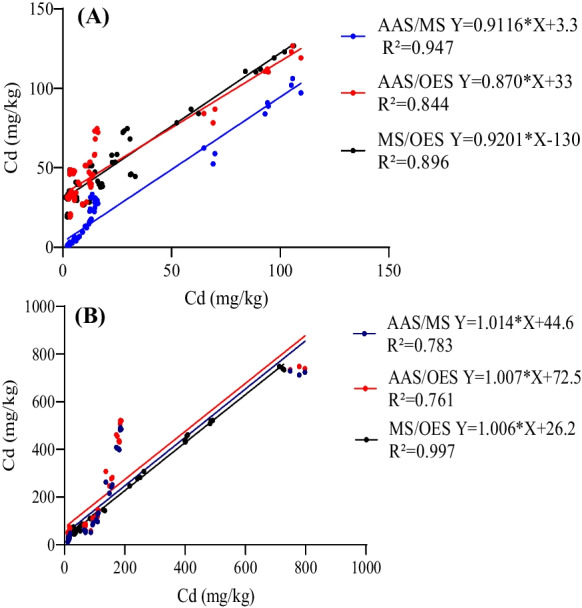
**A** Linear regression analysis of ramie samples under 0–50 mg/kg Cd treatment. **B** Linear regression analysis of ramie samples under 50–150 mg/kg cadmium treatment

As mentioned above, the values measured by GF-AAS were similar to the values measured by ICP-MS under 0, 10, 30, and 50 mg/kg concentrations of Cd treatment, and the result of the correlation analysis also conformed to these. The data measured by GF-AAS was lower than the data measured by ICP-MS and ICP-OES when samples were treated with 50, 80, and 150 mg/kg concentrations of Cd, which was also confirmed by the results of the correlation analysis. Here, the results of linear regression analysis further confirmed these results. As shown in Fig. 5A, the agreement between Cd concentrations determined by ICP-MS and GF-AAS was remarkably good, which was pointed out by a high value of R^2^ (0.947). It was concluded that it was a much better choice to use ICP-MS and GF-AAS to determine Cd content when samples were treated with low concentrations of Cd (0–50 mg/kg). As shown in Fig. 5, the data determined by ICP-MS was consistent with that determined by ICP-OES.

These two methods had a stronger relationship (R^2^=0.997) than the relationship between ICP-OES and GF-AAS (R^2^=0.761), and also stronger than the relationship between GF-AAS and ICP-MS (R^2^=0.783). It was concluded that using ICP-MS and ICP-OES to determine Cd content was more accurate than GF-AAS when samples were treated with high concentrations of Cd (80–150 mg/kg). In fact, the instrument for ICP-OES determination was more expensive than that of ICP-MS. Therefore, it is an advisable choice for us to choose ICP-MS when the detected sample has a high Cd content. GF-AAS wastes more time and sometimes you have to dilute the solution. Meanwhile, ICP-MS measurement not only saves time but also simultaneously detects multiple elements. Thus, it is a better choice to use ICP-MS than GF-AAS if your samples are at a lower Cd concentration level.

### Cost of Cd determination measured by GF-AAS, ICP-MS, and ICP-OES

To evaluate the cost of Cd determination detected by GF-AAS, ICP-MS, and ICP-OES, time and consumed materials were calculated (Table 7). The test time for ICP-MS and ICP-OES was 2–3 min per each sample, while it was 3–5 min for GF-AAS. Before Cd determination, the sample solution was diluted when using GF-AAS to measure. Thus, using GF-AAS to measure Cd content was time-consuming. However, argon was used when using ICP-MS and ICP-OES for Cd content, which increased the test cost. Moreover, the prices of the instruments for ICP-MS and ICP-OES measurement were much more expensive than that for GF-AAS measurement. In this point, there was a high testing cost when using ICP-MS and ICP-OES to measure Cd content. Using GF-AAS to detect Cd content wasted more time, while ICP-MS not only saved time but can also simultaneously detect multiple elements. Thus, it was a better choice of using ICP-MS than GF-AAS to detect the samples with a low Cd content.

**Table 7:** The cost of Cd content determination detected by ICP-OES, ICP-MS, and GF-AAS

Method	Price of the instrument	Diluted	Test time/sample	Consumed materials
GF-AAS	18 million RMB	YES	3~5 min	Tubes
ICP-MS	25 million RMB	NO	2~3 min	Argon and tubes
ICP-OES	48 million RMB	NO	2~3 min	Argon and tubes

## Discussion

Remediation of Cd pollution is one of the priorities of global environmental governance in heavy metal–polluted areas. Accurate detection of Cd content is a key link in remediation of Cd pollution. ICP-MS, GF-AAS, and ICP-OES were used to detect Cd content in blood or soil (McBride, 2011; Trzcinka-Ochocka et al., 2016). ICP-OES, GF-AAS, and ICP-MS are widely used and cost-effective, but which one is the best way to determine Cd and which one is faster and more accurate should be discussed. Previously, there were several documents about the comparison of ICP-MS, GF-AAS, and ICP-OES (Zeiner & Steffan, 2007; Zhu et al., 2011). However, only a few samples were used for analysis in these researches. Here, we used 162 ramie samples of various tissues from treatments of different Cd concentrations to compare the methods of ICP-MS, GF-AAS, and ICP-OES, and the differences among the three methods were discussed. We found that the GF-AAS, ICP-MS, and ICP-OES methods were all suitable for determination of Cd in ramie. In particular, ICP-MS and ICP-OES were simpler, faster, and more sensitive than the GF-AAS, while the GF-AAS was lower cost but more time-consuming than the other methods.

Previously, researches showed that ICP-OES and ICP-MS measurements were unable to quantify soil Cd at low near-background levels at the emission light of 226.5 nm, and it may provide biased values when Cd is at higher levels (McBride, 2011). In our study, we used the emission light of 228.8 nm in Cd determination, which was considered to be interfered by severe As or Ni concentration (McBride, 2011). For ICP-MS, if oxide or hydroxide ions of Pd, Sn, In, Zr, Mo, Ru, Nb, or Y are present in the sample at concentrations at least several orders of magnitude higher than the Cd concentration, there will be spectral interferences (May & Wiedmeyer, 1998). However, in this study, the Cd content in the samples was much higher than other elements, so that the interfering elements in these samples were not high enough to produce a false positive reading for Cd. Due to this problem, ICP-MS is the best suitable method to measure Cd content sample.

## Conclusions

In this study, we compared the three methods of ICP-OES, ICP-MS, and GF-AAS for Cd content determination using various ramie varieties for the first time. We recommend that ICP-MS was applicable to samples with various concentrations of Cd, and ICP-OES could be used for measurement of samples with > 100 mg/kg Cd content, while GF-AAS was suitable for the detection of samples with very high (> 550 mg/kg) or very low (< 10 mg/kg) Cd content. However, it was considered that using ICP-OES to measure may be affected by spectral interference and the instrument was expensive. Meanwhile, ICP-MS has shown good accuracy in both high and low concentrations of Cd determination. Therefore, we concluded that ICP-MS was the best suitable method to measure Cd content of ramie samples among the three methods.

## Supplementary information

ESM 1 (DOCX 19 kb)

## Data Availability

All data were included in the manuscript.
