# Supplementary material for: A comparative study of different methods for the determination of cadmium in various tissues of ramie (Boehmeria nivea L.)
Source: Environ Monit Assess. 2023 Jul 31;195(8):1009. doi: 10.1007/s10661-023-11601-2 (PMC10390602; doi:10.1007/s10661-023-11601-2)
Supplement: Supplementary file 1 — (DOCX 19 kb) [file 10661_2023_11601_MOESM1_ESM.docx]

**Table S1.** Program of microwave digestion.

| Step | Run time (min) | Hold time (min) | Control temperature (℃) | Power (W) |
| --- | --- | --- | --- | --- |
| 1 | 6 | 3 | 120 | 1600 |
| 2 | 6 | 10 | 150 | 1600 |
| 3 | 7 | 20 | 185 | 1600 |

**Table S2.** Typical operating parameters of ICP-OES.

| Parameter | Setting |
| --- | --- |
| Viewing mode | Axial |
| RF power /kW | 1.20 |
| Plasma Ar flow rate /(L/min) | 10.00 |
| Pneumatic nebulizer Ar flow rate /(L/min) | 0.70 |
| Auxiliary Ar flow rate /(L/min) | 0.60 |
| Read time /s | 30.00 |
| Rinse time /s | 20.00 |
| Elements wavelengths (nm) | Cd (226.50nm) |
| Number of replicates | 3 |

**Table S3.** The operating settings of ICP-MS.

| Parameter | Setting |
| --- | --- |
| RF power /kW | 1.20 |
| Sampling Cone /mm | 150.00 |
| Cooling gas /(L/min) | 18.00 |
| Auxiliary gas /(L/min) | 1.20 |
| Sampling gas /(L/min) | 0.85 |
| Liquid argon pressure /Mpa | 0.60 |
| Sample time /s | 40.00 |
| Wash time /s | 5.00 |
| Number of replicates | 3 |
| Internal standard element | Rh Ge In |

**Table S4.** Graphite furnace program of GF-AAS.

| Step | Temperature/℃ | Time/s | Slope（℃/s) | Type of gas | Gas flow（L/min) |
| --- | --- | --- | --- | --- | --- |
| step 1 | 100 | 30 | 10 | inert gas | 0.20 |
| step 2 | 600 | 20 | 150 | inert gas | 0.20 |
| step 3 | 1400 | 3.0 | 0 | inert gas | off |
| step 4 | 2500 | 3.0 | 0 | inert gas | 0.20 |
